# Cancer testis antigen MAGEA3 in serum and serum-derived exosomes serves as a promising biomarker in lung adenocarcinoma

**DOI:** 10.1038/s41598-024-58003-z

**Published:** 2024-03-30

**Authors:** Yuhan Gan, Yanli Kang, Ruifang Zhong, Jianbin You, Jiahao Chen, Ling Li, Jinhua Chen, Liangyuan Chen

**Affiliations:** Department of Clinical Laboratory, Shengli Clinical Medical College of Fujian Medical University, Fuzhou, China

**Keywords:** MAGEA3, Lung adenocarcinoma, Cancer-testis antigen, Exosome, Diagnosis, Tumor immune infiltration, Cancer, Immunology, Biomarkers

## Abstract

Cancer testis antigen (CTA) Melanoma Antigen Gene A3 (MAGEA3) were overexpressed in multiple tumor types, but the expression pattern of MAGEA3 in the serum of lung adenocarcinoma (LUAD) remains unclear. Clinically derived serum and serum exosome samples were used to assess the mRNA expression of *MAGEA3* and *MAGEA4* by qRT-PCR, and serum MAGEA3 and MAGEA4 protein expression were evaluated by ELISA in total 133 healthy volunteers’ and 289 LUAD patients’ serum samples. An analysis of the relationship of the mRNA and protein expression of MAGEA3 and MAGEA4 with clinicopathologic parameters was performed and the diagnostic value of MAGEA3 and MAGEA4 was plotted on an ROC curve. In addition, the correlation of *MAGEA3* mRNA with infiltrating immune cells was investigated through TIMER, the CIBERSORT algorithm and the TISIDB database. Expression of serum and serum exosome *MAGEA3* and *MAGEA4* mRNA were significantly higher in LUAD patients than in healthy donors. *MAGEA3* mRNA associated with tumor diameter, TMN stage, and NSE in LUAD serum samples, and *MAGEA3* mRNA correlated with N stage in serum-derived exosomes, possessing areas under the curve (AUC) of 0.721 and 0.832, respectively. Besides, serum MAGEA3 protein levels were elevated in LUAD patients, and were closely related to stage and NSE levels, possessing AUC of 0.781. Further analysis signified that the expression of *MAGEA3* mRNA was positive correlation with neutrophil, macrophages M2, dendritic cells resting, and eosinophilic, but negatively correlated with B cells, plasma cells, CD8 + T cells, CD4 + T cells, Th17 cells, macrophages and dendritic cells. Collectively, our results suggested that the MAGEA3 expression in mRNA and protein were upregulated in LUAD, and MAGEA3 could be used as a diagnostic biomarker and immunotherapy target for LUAD patients.

## Introduction

Lung cancer is one of the most frequently diagnosed tumors worldwide with approximately 2.2 million new cases and 1.79 million deaths annually^1^. Among them, lung adenocarcinoma (LUAD) is the leading subtype of lung cancer, accounting for 40% to 50% of lung cancer patients^2^. The majority of lung cancer patients were diagnosed at intermediate to advanced stages, losing time for optimal treatment^3^. Conventional screening method X-ray and low-dose chest-computed tomography scans have false positives, which may prompt patients to be over-treated^4^. Further, traditional tumor biomarkers like Neuron-Specific Enolase (NSE), Cytokeratin 19 Fragment (CYFRA21-1) and Carcinoembryonic Antigen (CEA) have no desired specificity and sensitivity^4,5^. Accordingly, a non-invasive biomarker for early diagnosis of LUAD is imperative.

Cancer testis antigens (CTAs) is a class of proteins that are restricted expression in germ cells of the testis and placenta but not expressed or under-expressed in other normal somatic cells^6^. Emerging evidence has shown that CTAs has abnormal expression when various oncogenesis^6,7^. The first identified members of the CTAs, Melanoma Antigen Gene (MAGE) family, is classified into MAGE-I and II according to their location and specific expression. MAGE-I located on the X chromosome includes MAGE-A, B and C subfamilies, while MAGE-II ubiquitous exist in healthy person includes others MAGE subfamily^8^. The MAGE-A family express abnormally in various tumors, including melanoma, lung cancer, breast cancer and pancreatic cancer^9–11^. Currently, growing studies concentrated on the MAGE-A family as a tumor biomarker, carcinogenesis and tumor immunotherapy target in multiple neoplasms^12,13^. MAGEA3 immunogenicity and carcinogenicity ranked eighth out of 75 tumor antigens in a National Cancer Institute study and ranked first in CTAs^14^. MAGEA4 is a prognostic biomarker in salivary gland carcinomas related to tumor grading^15^.The expression level of MAGEA 1–4, 6 and 12 increased when malignant transformation occurs in oral leucoplakia^16^. Besides, MAGE-A family can predict the effect of cytotoxic T lymphocyte-associated protein-4(CTLA-4) blockers in metastatic melanoma^17^. Nonetheless, the comprehensive analysis of the MAGE-A family in serum mRNA and protein level of LUAD remains unknown.

Exosomes are a subset of extracellular vesicles with a diameter range of 40-150 nm and involve in cell-to-cell communication by transporting their contents (nucleic acids, lipids, proteins) to target cells^18^. Exosomes can protect their "cargo" from RNase degradation in multiple body fluids due to their unique bilayer membranes, their contents have higher stability and a powerful potential as a diagnostic biomarker than serum^19,20^. MenXD et al. observed elevated levels of PLA2G10 mRNA and protein in serum exosomes, which can be a diagnostic marker to distinguish healthy from non-small cell lung cancer^21^. Thus, exploring particular mRNA in serum exosomes as a biomarker for early diagnosis of LUAD is worthwhile.

In the present study, we firstly screened potential biomarkers in serum MAGEA1-6, and then the database-derived tissues and clinic-derived serum expression levels of MAGEA3 and MAGEA4 were investigated in LUAD. Finally, the correlation between MAGEA3 expression and immune cells infiltrating levels were explored. Our results uncovered the significant function of MAGEA3 in LUAD, as well as proposed potential connection between MAGEA3 and immune infiltration of LUAD patients.

## Materials and methods

### Clinical samples

Serums were collected from 289 patients with lung adenocarcinoma and 133 healthy volunteers from the Clinical Laboratory, Fujian Provincial Hospital (Fuzhou, China) between January 2020 and March 2023. Lung adenocarcinoma was diagnosed via pathological analysis. All the donors had signed the informed consent, and none was treated with radiotherapy or chemotherapy prior to collection. Clinicopathological characteristics were recorded, including age, gender, tumor diameter, TNM classification, stage etc. The present study was conducted with the approval of the ethics committee of Fujian Provincial Hospital and complied with the ethical standards of the Helsinki Declaration.

### RNA isolation and quantitative real-time polymerase chain reaction (qRT‑PCR)

We used TRIzol™ LS Reagent (Invitrogen, Carlsbad, CA, USA) to extract the total RNA from 254 serum samples based on the manufacturer’s instructions. Then, RNA was dissolved in enzyme-free water and the concentration was quantified by NanoDrop One/Onec Spectrophotometer (Thermo Scientific). The PrimeScript™ RT reagent kit (RR037A; TAKARA, Dalian, China) was used for reversing transcription under 37 °C for 15 min, followed by 85 °C for 5 s. Finally, cDNA was amplified by TB Green^®^ Premix Ex Taq™ II Kit (RR820A; TAKARA, Dalian, China), following the manufacturer’s instruction in 40 cycles of denaturation at 95 °C for 10 min, 95 °C for 15 s, with extension at 60 °C for 1 min using LightCycler 480 System. The 2^−ΔΔCT^ method indicated the relative expression level, and GAPDH served as a reference gene. QRT-PCR’s primer sequences are shown in Table S1.

### Enzyme linked immunosorbent assay (ELISA)

According to the manufacturer’s protocol, the serum protein of MAGEA3 and MAGEA4 was detected by the Human ELISA Kit (MLBio, Shanghai, China). Diluting standards at the concentrations indicated in the manufacturer’s instructions for determination of samples’ concentration. Serum samples were diluted at 1:5, which added 10μL of serum to 40μL of diluent and incubated at 37 °C for 30 min. After washing the plate with phosphate-buffered saline with Tween 20 (PBST), 50μL of HRP conjugated reagent was added and incubated at 37 °C for 30 min. 50μL of chromogen solution A and B were then added to each well in the dark and incubated for 10 min at 37 °C. Finally, 50μL of the stopping solution was added, and OD values were measured within 15 min at 450 nm wavelength using an enzyme-labelled instrument (Bio-Rad).

### The Cancer Genome Atlas (TCGA) database

The gene expression profiles of LUAD patients were obtained from the TCGA database (http://portal.gdc.cancer.gov/). We analyzed the expression of *MAGEA3* and *MAGEA4* mRNA between 541 LUAD samples and 59 adjacent para-cancerous lung tissues. Besides, the expression levels of MAGEA3 and MAGEA4 were further compared between 59 LUAD tissues and matched normal tissues using TCGA database.

### The Kaplan–Meier plotter database

The Kaplan–Meier plotter database contained survival information for 865 patients with LUAD (http://kmplot.com)^22^. LUAD patients were divided into high expression group and low expression group according to the median expression level of MAGEA3 or MAGEA4. The prognostic value of MAGEA3 and MAGEA4 (progression free survival) was assessed by Kaplan–Meier plotter.

### PrognoScan database

The correlation between MAGEA3 and MAGEA4 expression and overall survival (OS) was explored by PrognoScan database, which is a freely available resource was collected from Gene Expression Omnibus (GEO), ArrayExpress, and individual laboratory websites (http://www.prognoscan.org/). Cox P value < 0.05 was considered statistically significant.

### TIMER database

TIMER is a user-friendly, comprehensive database containing over 10,000 tumor samples from TCGA for 32 cancer types (https://cistrome.shinyapps.io/timer/)^23^. In the present study, we used TIMER to estimate the relationship between MAGEA3 expression and immune infiltrates of B cells, CD4 + T cells, CD8 + T cells, neutrophils, macrophages, and dendritic cells in LUAD.

### CIBERSORT algorithm

CIBERSORT (https://www.biostars.org/p/428905/) is an analytical tool, which aids in estimating the proportions of 22 types of infiltrating immune cells through gene expression data^24^. We explored the relationship between MAGEA3 and tumor-infiltrating immune cells using CIBERSORT algorithm.

### TISIDB database

TISIDB (http://cis.hku.hk/TISIDB/) is an online portal for tumor and immune system interaction^25^. TISIDB was used to examine MAGEA3 and tumor-infiltrating cells expression in LUAD.

### Statistical analysis

R software package was used to implement TCGA database analysis. The statistical analyses were performed with the SPSS 25.0 software package (SPSS Inc. Chicago, USA) and GraphPad Prism 9.0 (GraphPad Software, USA). Survival curves were generated using Kaplan–Meier plots. QRT-PCR and ELISA were analyzed using the unpaired Student’s t-test. The receiver operating characteristic (ROC) curve and the area under the curve (AUC) were used to analyze the diagnostic efficiency. Spearman test was used to measure the correlation between MAGEA3 and Tumor infiltrating lymphocytes (TILs). When P < 0.05, the data is statistically significant.

### Ethical approval

This study was performed in line with the principles of the Declaration of Helsinki. Approval was granted by the Ethics Committee of Fujian Provincial Hospital.

### Consent to participate

Informed consent was obtained from all individual participants included in the study.

## Results

### MAGEA1-6 expression in LUAD

We first conducted a preliminary experiment using qRT-PCR to explore potential targets of MAGEA1-6 in lung adenocarcinoma by 60 samples. Due to MAGEA5 from house mouse, it was except (https://www.ncbi.nlm.nih.gov/gene/17141). The results showed that MAGEA3 and MAGEA4 had a statistical significance (P < 0.05), but MAGEA1, MAGEA2 and MAGEA6 had no statistical significance (P > 0.05) (Table S2). Based on this, we chose MAGEA3 and MAGEA4 for further exploration.

### Aberrant expression and prognostic value of MAGEA3 and MAGEA4 in LUAD

**Figure 1:**
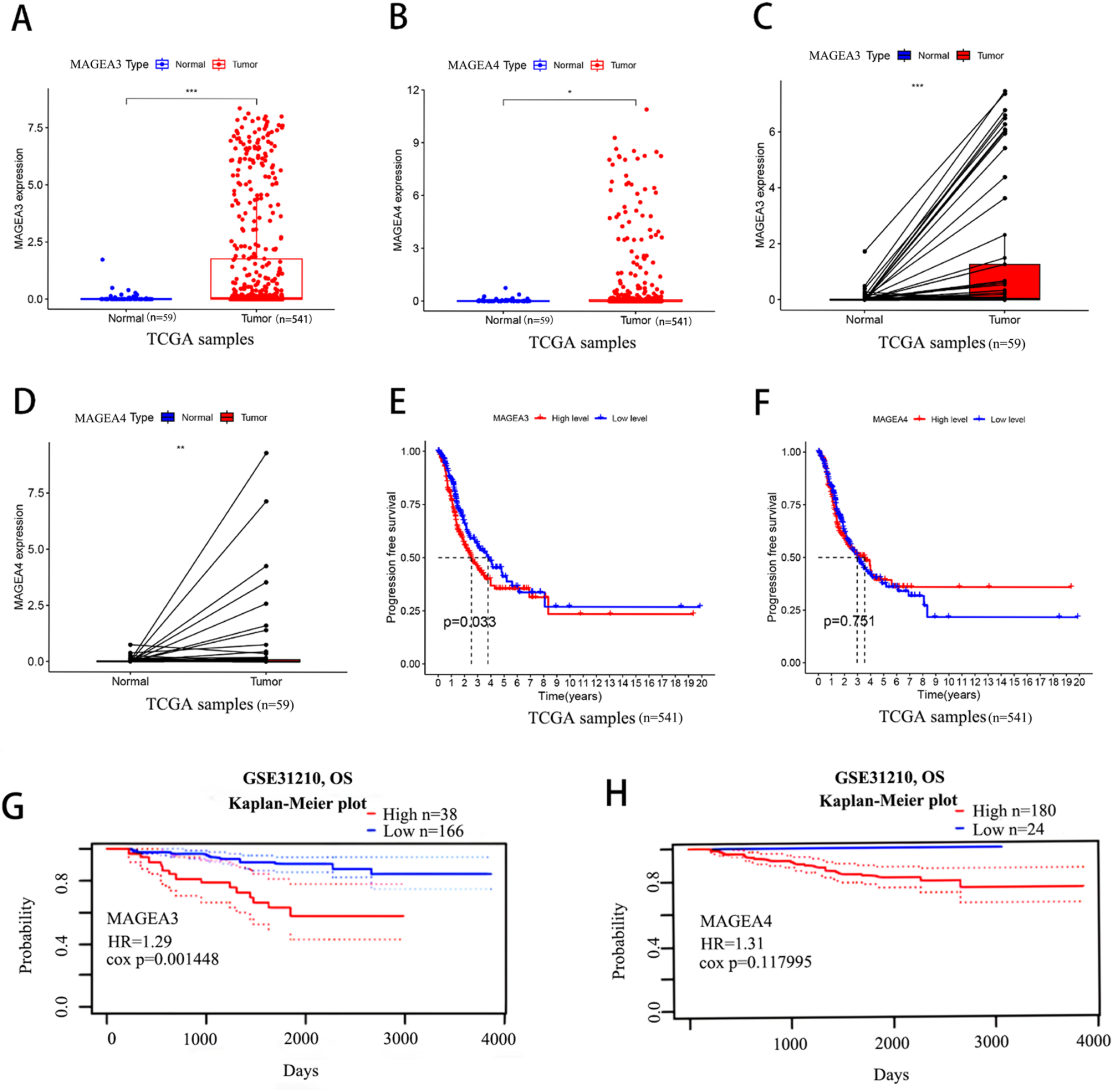
Expression levels and prognosis of MAGEA3 and MAGEA4 in LUAD patients. The expression levels of MAGEA3 and MAGEA4 in LUAD and para-cancerous lung tissues by TCGA database (**A**,**B**). MAGEA3 and MAGEA4 expression in LUAD and matched para-carcinoma tissue by TCGA database (**C**,**D**). The progression free survival (PFS) rate of MAGEA3 and MAGEA4 expression in TCGA LUAD samples (**E**,**F**). The overall survival (OS) of MAGEA3 and MAGEA4 expression in GSE31210 cohorts of PrognoScan database (**G**,**H**).

To further corroborate the preliminary experimental results, we analyzed the gene expression profiles of 541 LUAD patients’ samples and 59 normal samples from the TCGA database. The analysis showed that MAGEA3 had a remarkable enhanced in LUAD samples than the adjacent normal lung tissues (Fig. 1A). The expression level of MAGEA4 was elevated in LUAD samples, too (Fig. 1B). The results were in line with the 59 matched tissue samples from the LUAD patients (Fig. 1C,D). After that, we analyzed the relationship between the expression level of MAGEA3 and MAGEA4 and prognosis in LUAD by Kaplan–Meier plotter and PrognoScan database. As show in Fig. 1E–H, high MAGEA3 expression was associated with a poor progression free survival (PFS) and overall survival (OS), while MAGEA4 had no correlation with prognosis(P > 0.05).

### Serum and serum exosome expression of MAGEA3 and MAGEA4 in LUAD

**Figure 2:**
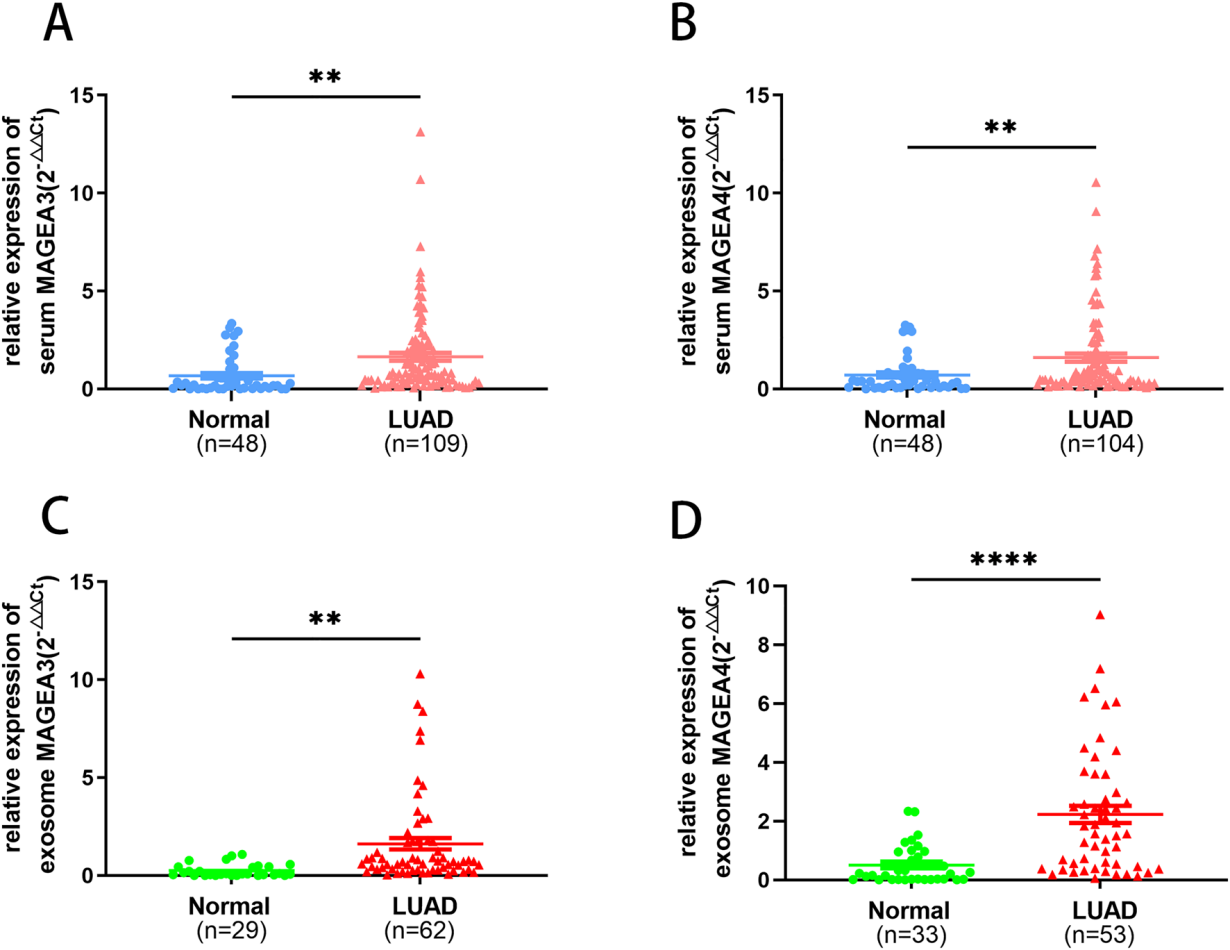
The expression of MAGEA3 and MAGEA4 in serum and serum-derived exosome with patients of LUAD patients using qRT-PCR. Serum mRNA expression of MAGEA3 (**A**) and MAGEA4 (**B**), and serum-derived exosome expression of MAGEA3 (**C**) and MAGEA4 (D). (**P < 0.01, ****P < 0.0001).

We expanded the sample size for exploration to verify the preliminary experimental results. The qRT-PCR results indicated that MAGEA3 was higher in 109 LUAD patients than in 48 healthy volunteers (P = 0.0024), and MAGEA4 increased in serum from 104 LUAD patients compared to 48 healthy volunteers (P = 0.0052) (Fig. 2A,B). The clinicopathological feature shed light into a solid relationship between MAGEA3 and TNM classification, stage and tumor diameter (P < 0.05) (Table 1).

**Table 1:** Correlation of serum *MAGEA3 and MAGEA4* mRNA expression and clinicopathological parameters in LUAD patients.

Clinicopathological factor	n	MAGEA3		n	MAGEA4	
		Mean ± SEM	P		Mean ± SEM	P
Age (years)			0.2448			0.5942
< 60	65	1.450 ± 0.1868		49	1.701 ± 0.2941	
≥ 60	44	1.924 ± 0.4083		45	1.478 ± 0.2822	
Gender			0.9123			0.7437
Male	41	1.670 ± 0.3602		41	1.689 ± 0.3287	
Female	68	1.624 ± 0.2359		63	1.550 ± 0.2664	
Diameter (cm)			**0.0337**			0.2076
< 2	83	1.353 ± 0.1818		76	1.621 ± 0.2418	
≥ 2	13	2.344 ± 0.6649		13	2.466 ± 0.7765	
Stage			**0.0421**			0.058
< II	91	1.462 ± 0.1827		84	1.795 ± 0.2477	
≥ II	18	2.548 ± 0.9686		20	0.806 ± 0.1714	
T			**0.0004**			0.0694
Tis + 1	90	1.391 ± 0.1500		79	1.815 ± 0.2544	
≥ 2	14	3.505 ± 1.1140		25	0.941 ± 0.2627	
N			**0.0009**			0.1353
0	93	1.443 ± 0.1595		86	1.783 ± 0.2428	
≥ 1	11	3.637 ± 1.3330		14	0.865 ± 0.1959	
M			**0.0124**			0.1100
0	97	1.467 ± 0.1585		91	1.730 ± 0.2307	
≥ 1	12	3.046 ± 1.2490		13	0.733 ± 0.2216	
CEA			0.3195			0.1556
< 5 ng/ml	87	1.590 ± 0.1997		83	1.737 ± 0.2459	
≥ 5 ng/ml	16	2.169 ± 0.8130		17	0.938 ± 0.2694	
NSE			0.0628			0.4731
< 16.3 ng/ml	63	1.471 ± 0.2057		59	1.481 ± 0.2960	
≥ 16.3 ng/ml	8	2.924 ± 1.4890		11	0.974 ± 0.3303	

Significant values are in bold.

*MAGEA3* melanoma-associated antigen family A3, *MAGEA4* melanoma-associated antigen family A4, *LUAD* lung adenocarcinoma, *Tis* tumor in situ, *TNM* tumor node metastasis, *CEA* carcinoembryonic antigen, *NSE* neuron-specific enolase.

Exosomes as a more stable cargo, we further extracted serum exosome successfully and investigated the expression levels of MAGEA3 and MAGEA4 in serum-derived exosomes by qRT-PCR. As shown in Fig. 2C and D, the expression of MAGEA3 (P = 0.0021) and MAGEA4 (P < 0.0001) in serum exosomes were high expression and MAGEA3 level was positively correlated with lymph node metastasis of LUAD patients (Table 2). Of note, the levels of MAGEA3 and MAGEA4 in serum exosomes were statistically higher than those in serum samples. Nevertheless, there were nonsignificant correlations between MAGEA4 of serum and serum-derived exosome and clinicopathological factor (Tables 1, 2). Thus, MAGEA3 and MAGEA4 were all abundant in LUAD patients’ serum and serum-derived exosome, and MAGEA3 was more clinically significant.

**Table 2:** Correlation of serum exosomal MAGEA3 and MAGEA4 expression and clinicopathological parameters.

Clinicopathological factor	n	MAGEA3 serum exosome		n	MAGEA4 serum exosome	
		Mean ± SEM	P		Mean ± SEM	P
Age(year)			0.1414			0.8863
< 60	35	1.153 ± 0.1895		30	2.138 ± 0.3521	
≥ 60	27	1.960 ± 0.5666		23	2.221 ± 0.4727	
Gender			0.1476			0.3117
Male	23	2.017 ± 0.5765		20	1.802 ± 0.4019	
Female	39	1.202 ± 0.2600		33	2.399 ± 0.3833	
Diameter			0.7723			0.6400
< 2	39	1.567 ± 0.3479		34	2.200 ± 0.3720	
≥ 2	15	1.768 ± 0.6551		13	2.529 ± 0.5716	
Stage			0.1492			0.8776
< II	42	1.233 ± 0.2610		39	2.200 ± 0.3499	
≥ II	20	2.073 ± 0.6307		14	2.100 ± 0.4659	
T			0.6924			0.2933
Tis + 1	45	1.472 ± 0.2919		40	2.374 ± 0.3567	
≥ 2	15	1.730 ± 0.7122		12	1.652 ± 0.3335	
N			**0.018**			0.8856
0	48	1.210 ± 0.2401		42	2.228 ± 0.3506	
≥ 1	12	2.842 ± 0.9551		10	2.121 ± 0.2623	
M			0.9807			0.6519
0	49	1.508 ± 0.3147		43	2.112 ± 0.3194	
≥ 1	13	1.491 ± 0.5380		10	2.442 ± 0.6310	
CEA			0.1858			0.6929
< 5 ng/ml	44	1.135 ± 0.2388		23	2.214 ± 0.4702	
≥ 5 ng/ml	13	1.868 ± 0.6309		25	1.972 ± 0.3949	
NSE			0.8648			0.9833
< 16.3 ng/ml	36	1.388 ± 0.3599		34	2.116 ± 0.4057	
≥ 16.3 ng/ml	7	1.245 ± 0.3410		5	2.093 ± 0.4284	

Significant values are in bold.

### Diagnostic value of MAGEA3 and MAGEA4 of LUAD in serum and serum exosome

**Figure 3:**
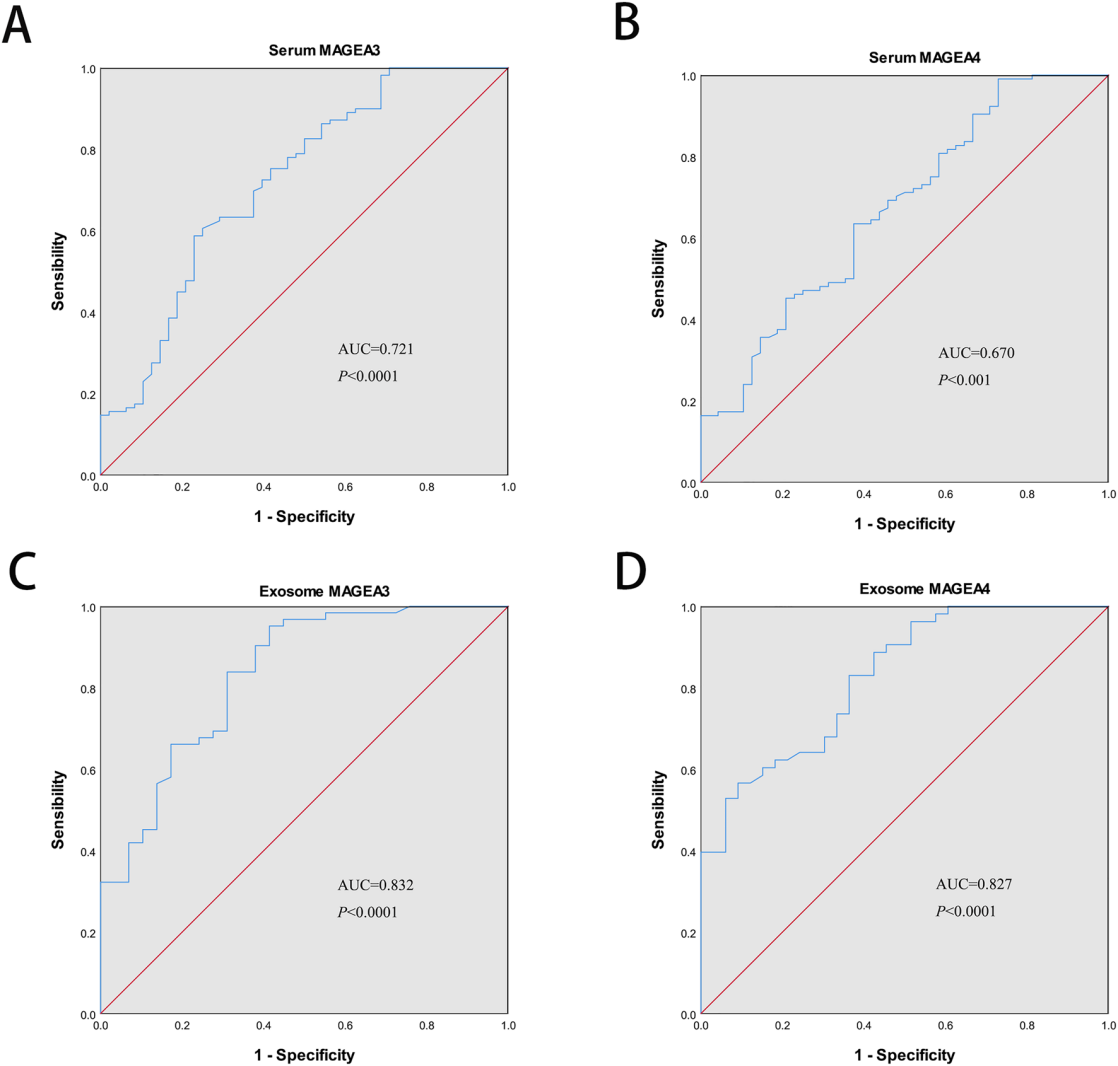
Diagnostic value of MAGEA3 and MAGEA4 in serum (**A**,**B**) and serum-derived exosome (**C**,**D**) of patients with LUAD by plotting receiver operating characteristic (ROC) curves.

Next, we assessed the diagnostic significance of MAGEA3 and MAGEA4 in serum and serum exosomes. For MAGEA3 mRNA expression, ROC curves analysis revealed that AUC of serum MAGEA3 was 0.721 with a sensitivity of 58.70%, specificity of 77.10%, and the AUC of serum exosome of MAGEA3 was 0.832 with a sensitivity of 95.20%, specificity of 58.60% (Fig. 3A,C and Table 3). Meanwhile, the ROC curves analysis yielded an AUC of 0.670 with sensitivity of 99.00% against specificity of 27.10% for the serum MAGEA4, and an AUC of 0.827 with sensitivity of 56.60% against specificity of 90.00% for the serum exosome MAGEA4 (Fig. 3B,D and Table 3). The MAGEA3 had superior AUCs than MAGEA4 regardless of serum or serum-derived exosome.

**Table 3:** Diagnostic efficacy of *MAGEA3* and *MAGEA4* mRNA in serum and serum exosome and diagnostic efficacy of MAGEA3 protein in serum.

Variables		AUC (95% CI)	Cut off	Sensitivity (%)	Specificity (%)
mRNA expression	Serum MAGEA3	0.721 (0.631–0.812)	0.358	58.70	77.10
	Serum MAGEA4	0.670 (0.577–0.764)	0.261	99.00	27.10
	Exosome MAGEA3	0.832 (0.741–0.923)	0.538	95.20	58.60
	Exosome MAGEA4	0.827 (0.741–0.913)	0.475	56.60	90.00
Protein expression	Serum MAGEA3	0.781 (0.686–0.876)	0.533	82.10	72.10

### Serum protein expression of MAGEA3 and MAGEA4 in LUAD

**Figure 4:**
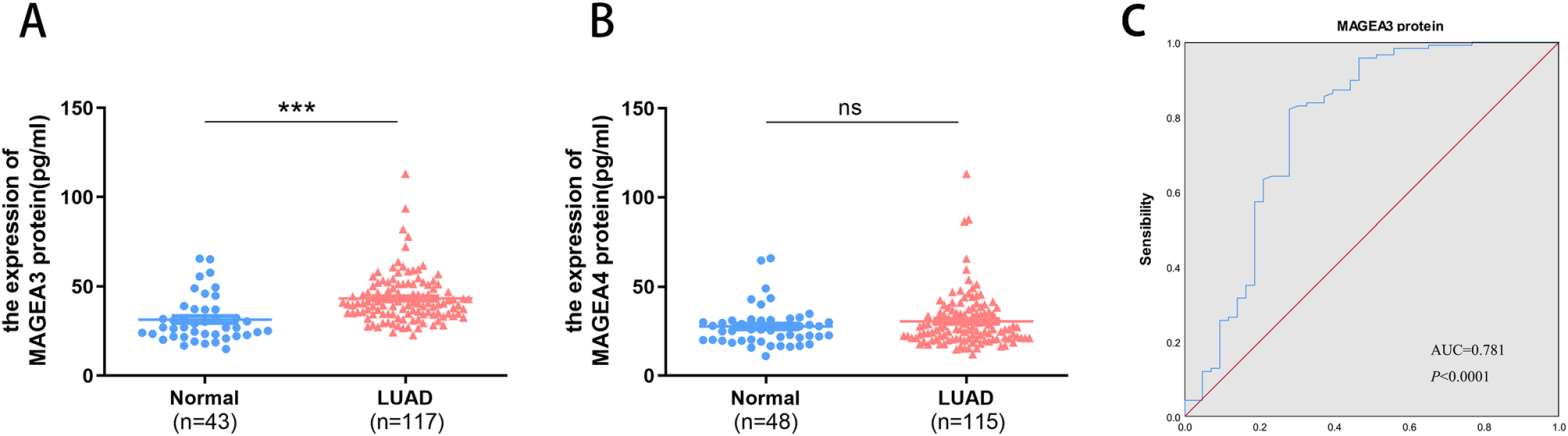
The expression of MAGEA3 and MAGEA4 protein in serum with patients of LUAD by ELISA. Protein expression of MAGEA3 (**A**) and MAGEA4 (**B**) in serum. Diagnostic performance of MAGEA3 protein (**C**) in serum by ROC curves. (ns P ≥ 0.05, ***P < 0.001).

We then evaluated the expression level of serous MAGEA3 and MAGEA4 proteins in LUAD by ELISA. The results revealed that serous MAGEA3 protein was remarkably elevated in 117 LUAD patients (P < 0.0001). The sensitivity, specificity, and the AUC of MAGEA3 protein were estimated to be of 82.10%, 72.10%, and 0.781 (p < 0.0001), respectively (Fig. 4A,C and Table 3). Furthermore, MAGEA3 had significant differences in the clinical stage and traditional tumor biomarker NSE (Table 4). Unfortunately, there was no difference in MAGEA4 protein expression between 115 LUAD patients and 48 healthy controls (P > 0.05) (Fig. 4B).

**Table 4:** Correlation of MAGEA3 protein expression and clinicopathological parameters.

Clinicopathological factor	n	MAGEA3 protein	
		Mean ± SEM	P
Age (year)			0.816
< 60	72	43.05 ± 1.569	
≥ 60	45	43.66 ± 2.137	
Gender			0.101
Male	47	45.82 ± 2.611	
Female	70	41.58 ± 1.151	
Diameter			0.6
< 2	75	43.74 ± 1.635	
≥ 2	19	41.88 ± 2.771	
Stage			**0.031**
< II	83	41.55 ± 1.187	
≥ II	34	47.52 ± 3.159	
T			0.11
Tis + 1	82	42.41 ± 1.468	
≥ 2	28	47.28 ± 2.879	
N			0.283
0	81	42.38 ± 1.503	
≥ 1	36	45.33 ± 2.322	
M			0.863
0	97	43.18 ± 1.384	
≥ 1	20	43.77 ± 3.167	
CEA			0.922
< 5 ng/ml	85	43.51 ± 1.503	
≥ 5 ng/ml	27	43.21 ± 2.685	
NSE			**0.013**
< 16.3 ng/ml	75	42.94 ± 1.455	
≥ 16.3 ng/ml	9	55.52 ± 7.764	

Significant values are in bold.

### Association between MAGEA3 and tumor infiltrating immune cells in LUAD

**Figure 5:**
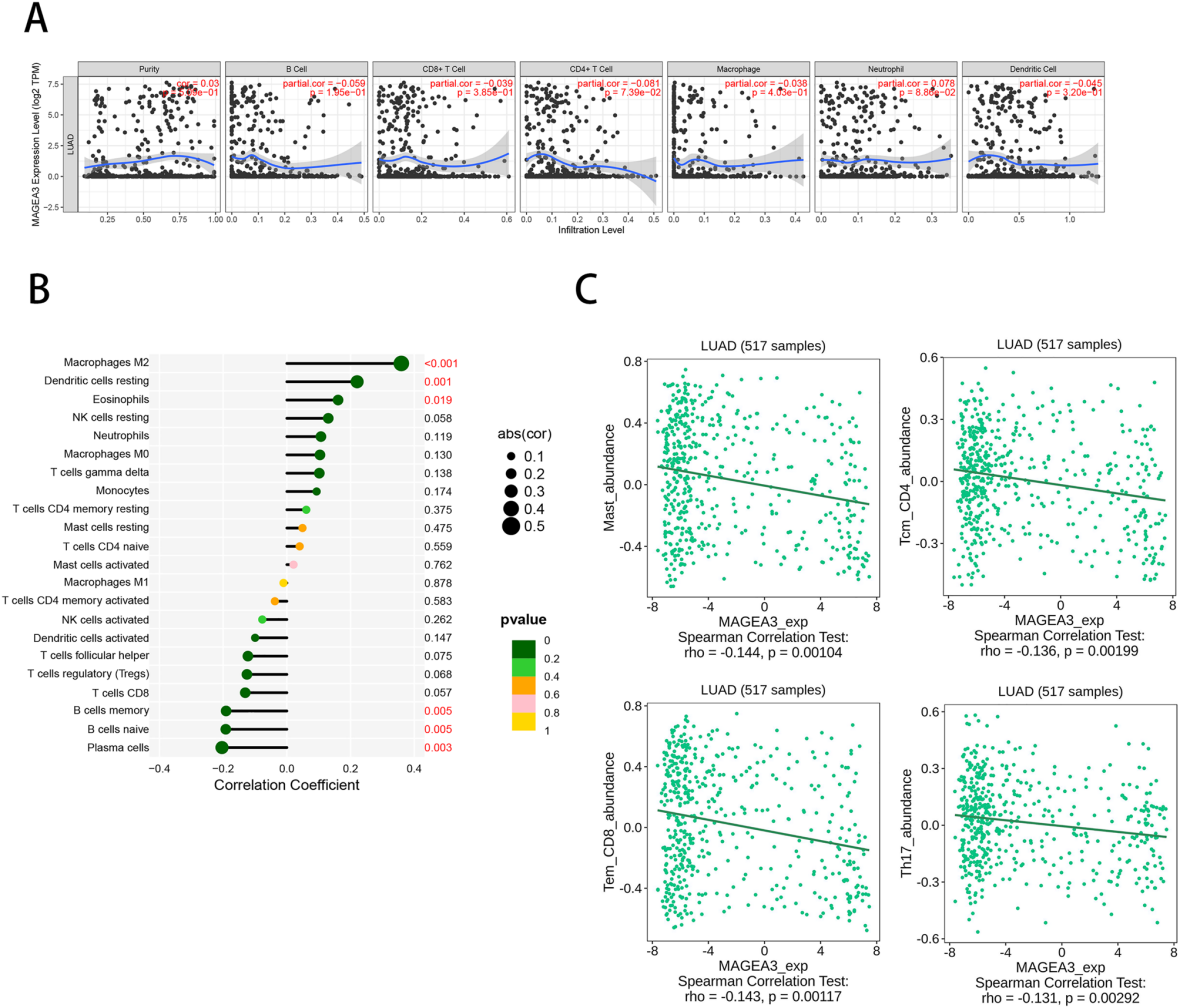
Correlation between MAGEA3 expression and immune infiltration in LUAD. The association between MAGEA3 and infiltration of purity, B cells, CD8 + T cells, CD4 + T cells, macrophage, neutrophil, dendritic cells was analyzed using TIMER (**A**). The relationship between MAGEA3 and the infiltrating abundance of different immune cells was investigated using CIBERSORT (**B**). The association between MAGEA3 and immune cells was analyzed by TISIDB (**C**).

CTA is suitable targets for cancer immunotherapy due to its unique immune-privileged characteristic^6^. Moreover, the ELISA results suggest that MAGEA3 is conspicuous associated with humoral immunity in LUAD. Therefore, we further explored the relationship between MAGEA3 and tumor immune cell infiltration. The TIMER analysis results showed that MAGEA3 expression had a positive correlation with neutrophil, and a negative correlation with B cells, CD8 + T cells, CD4 + T cells, macrophages and dendritic cells (Fig. 5A). Furthermore, high expression of MAGEA3 is associated with infiltration of macrophages M2, dendritic cells resting, and eosinophilic, as well as decreased expression of B cells memory, B cells naive, and plasma cells by CIBERSORT algorithm (Fig. 5B). This negative correlation between the expression level of MAGEA3 and central CD4 + T cells, CD8 + T cells and Th17 cells using TISIDB database (Fig. 5C).These results indicated that MAGEA3 might have an important effect on tumor immune infiltration in LUAD.

## Discussion

The Melanoma-Associated Antigen Family A (MAGE-A), which is the earliest Cancer-Testis Antigens (CTAs) identified in melanoma as tumor specific antigens, abnormally expressed in a variety of malignant tumors, including lung cancer^26^. Several studies have investigated the expression of MAGE-A family in NSCLC tissues and lung cancer cell lines using IHC or RT-PCR^27–29^. Considering that these serum markers have the advantage of being readily obtained, calculated and inexpensive, they may become of considerable value. Blood have been chosen as compartment for the prediction of broadly disseminated disease and systemic tumor load. However, to the best of our knowledge, the mRNA and protein expression levels of MAGE-A family in serum of LUAD patients have not yet been investigated.

It was reported that different members of the MAGE-A gene family are abnormally expressed in lung cancer tissues and those studies that do exist are each individually focused on a small subset of *MAGE-A* genes^30^. Our result showed that in the comprehensive analysis of MAGE-A1-6 expression, MAGEA3 and MAGEA4 were significant differentially expressed in serum of LUAD patients. And we further validated their expression and prognosis by analyzing LUAD tissues from databases derived. In the study of the differential expressed profile of the different *MAGE* genes subclass in NSCLC tumor tissue, 70% of samples expressed MAGE-A1 and 85% expressed MAGEA3^31^. Lina et al. qualitatively analyzed the expression of MAGEA1-6 in the peripheral blood of 150 lung cancer patients using multiplex semi-nested PCR, and the positive rate was MAGEA2(15.3%) > MAGEA6 > MAGEA4 > MAGEA3 > MAGEA1^27^. In fact, the frequency of CTAs expression was variable depend on histologic subtypes, which might account for the difference frequency of expression of MAGE-A family between our study and previous studies^32,33^.

We analyzed the expression of MAGEA3 and MAGEA4 in the serum and serum exosome of LUAD patients using qRT-PCR. MAGEA3 and MAGEA4 were up-regulated in LUAD. These results paralleled similar findings in previous studies, indicated MAGEA3 and MAGEA4 may serve as a diagnostic and prognostic biomarker for patients with NSCLC^28,29^. It’s not surprising that the diagnostic efficacy of serum exosome MAGEA3 and MAGEA4 was better than that of serum, and the AUC was higher than 0.8. As one of the media of information communication between cells, exosomes present a lipid bilayer, which gives them stability in the bloodstream or during bulk storage, preserving their content against degradation^18^. To investigate if *MAGE-A* mRNA expression is translated into MAGE-A protein, our study was the first time to analyze the serum protein level of MAGEA3 and MAGEA4 using ELISA. The interesting thing is that the expression level of serum MAGEA3 protein was elevated in LUAD patients than healthy people, but MAGEA4 was meaningless. Furthermore, the protein expression of MAGEA3 correlated with stage and tumor biomarker NSE, and the diagnostic efficacy of MAGEA3 serum protein is better than that of serum. In Cai et al. study, 8 autoantibodies targeting tumor-associated antigens (TAAs) were obtained via liquid chip technique, including MAGEA4 but no MAGEA3^34^. We hypothesized that autoantibody production is sufficient to neutralize detection of the MAGEA4 protein in serum. As we known, clinical pathological parameters, especially TNM staging, are closely related to the prognosis of patients. In contrast to MAGEA3, MAGEA4 was not associated with the clinical pathological parameters of LUAD patients. This suggests that there is little correlation between MAGEA4 and clinical progression in patients with LUAD. In addition, several studies in MAGEA3 peptide vaccines have been used in patients with NSCLC^35,36^. These results suggest that MAGEA3 is the best biomarker of MAGEA1-6 in LUAD.

MAGEA3 are antigens encoded by cancer-germline genes, and have been identified as a potential prognostic biomarker and pro-survival factor in multiple types of cancer^37–39^. The expression of CT genes is known to be regulated epigenetically by promoter methylation. MAGEA3 expression levels were closely associated with markers of active histone modifications in breast cancer cell^40^. Brother of the regulator of imprinted Sites (BORIS) was reported that it bound to the promoters of *MAGEA3* genes and was associated with their transcriptional activation in lung cancer^29^. These above reports might explain the phenomena we observed here at high expression levels of MAGEA3 in serum and serum exosomes of LUAD patients and also promised that MAGEA3 is the most important tumor antigen target of MAGEA1-6 in LUAD. As CTAs are known to be immunogenic, we finally explored the relationship between the high level of MAGEA3 and immune cell infiltration in LUAD. In our study, we found that MAGEA3 was negative correlated with CD8 + cells, CD4 + cells, Th17, B cell, plasma cells and dendritic cell, which are related to humoral and cellular immunity. Kim et al. also reported that the high expression of MAGEA3 lead to infiltrate of less dendritic cells, which lead to tumor cells escape the immune surveillance of a host^41^. Macrophages M2, a cancer-promoting phenotype of macrophages, was positive correlated with MAGEA3, which indicated that with the increase of MAGEA3, it contributes to the formation of tumor microenvironment promoting cancer. In a word, the correlation between MAGEA3 and immune cells further demonstrated that MAGEA3 seemed to play an important role in oncogenesis. The immunotherapy related to MAGEA3 in lung cancer has entered the clinical trial stage, and the immunogenicity and safety are good^42,43^. Along this line, it is perhaps more plausible that the improved immunotherapy response in anti-MAGEA3 immunization trials were mediated through humoral and cellular immunological mechanisms.

To sum up, the mRNA expression levels of *MAGEA3* and *MAGEA4* in tissues, serum and serum exosomes of LUAD patients were elevated and correlated with poor prognosis. The expression of MAGEA3 in serum and serum exosome were closely related to clinicopathological parameters. MAGEA3 is still highly expressed in serum proteins of LUAD patients, which is related to stage and NSE. In addition, the expression of MAGEA3 correlated with the infiltration of immune lymphocytes. Our results suggested that the mRNA and protein expression of MAGEA3 were upregulated in LUAD, and MAGEA3 could be used as a diagnostic biomarker and immunotherapy target for LUAD patients. However, the immune mechanism needs further studied.

### Supplementary Information

Supplementary Table S1.

Supplementary Table S2.

## Data Availability

The datasets generated during and/or analyzed during the current study are available from the corresponding author on reasonable request.
