# Supplementary material for: Cancer testis antigen MAGEA3 in serum and serum-derived exosomes serves as a promising biomarker in lung adenocarcinoma
Source: Sci Rep. 2024 Mar 30;14:7573. doi: 10.1038/s41598-024-58003-z (PMC10981702; doi:10.1038/s41598-024-58003-z)
Supplement: Supplementary file 1 — Supplementary Table S1. [file 41598_2024_58003_MOESM1_ESM.docx]

Table S1. Primer sequences for quantitative real-time polymerase chain reaction.

| Symbol | Primer sequences(5'-3') |
| --- | --- |
| GAPDH | F:TGACTTCAACAGCGACACCCA |
|  | R:CACCCTGTTGCTGTAGCCAAA |
| MAGEA1 | F:CGGCCGAAGGAACCTGACCCAG |
|  | R:GCTGGAACCCTCACTGGGTTGCC |
| MAGEA2 | F:AAGTAGGACCCGAGGCACTG |
|  | R:GAAGAGGAAGAAGCGGTCTG |
| MAGEA3 | F:AGAAGATCTGCCAGTGGGTC |
|  | R:CTGCCAATTTCCGACGACAC |
| MAGEA4 | F:GAAGATCTGCCTGTGGGTCC |
|  | R:GAGAGGAGGAGGAGACAGCA |
| MAGEA6 | F:GTCGTCGGAAATTGGCAGT |
|  | R:GCAGGTGGCAAAGATGTACAC |
