# Supplementary material for: Cancer testis antigen MAGEA3 in serum and serum-derived exosomes serves as a promising biomarker in lung adenocarcinoma
Source: Sci Rep. 2024 Mar 30;14:7573. doi: 10.1038/s41598-024-58003-z (PMC10981702; doi:10.1038/s41598-024-58003-z)
Supplement: Supplementary file 2 — Supplementary Table S2. [file 41598_2024_58003_MOESM2_ESM.docx]

| Symbol |  | Normal |  | LUAD | P |
| --- | --- | --- | --- | --- | --- |
|  | n | (Mean±SEM) | n | (Mean±SEM) |  |
| MAGEA1 | 7 | 1.334 ±0.5186 | 12 | 0.667 ±0.1188 | 0.1289 |
| MAGEA2 | 7 | 4.378 ±2.0060 | 12 | 16.05 ±6.3440 | 0.1897 |
| MAGEA3 | 7 | 0.457 ±0.1127 | 12 | 3.584 ±0.9234 | 0.0207 |
| MAGEA4 | 7 | 0.484 ±0.1157 | 12 | 1.890 ±0.3144 | 0.0042 |
| MAGEA6 | 7 | 3.253 ±1.0390 | 12 | 4.346 ±2.0640 | 0.7055 |

Table S2. *MAGEA1*, *MAGEA2*, *MAGEA3*, *MAGEA4* and *MAGEA6* mRNA levels in LUAD patients’ serum by qRT-PCR.
